# Correction: Performance under pressure in skill tasks: An analysis of professional darts

**DOI:** 10.1371/journal.pone.0230528

**Published:** 2020-03-12

**Authors:** The PLOS ONE Staff

The Data Availability statement for this paper is incorrect. The correct statement is: An anonymized version of the dataset and the corresponding R-Code are uploaded to GitHub (https://github.com/marius-oetting/performance-under-pressure-in-darts). The publisher apologizes for the error.

## References

[1] Ötting M, Deutscher C, Schneemann S, Langrock R, Gehrmann S, Scholten H (2020) Performance under pressure in skill tasks: An analysis of professional darts. PLoS ONE 15(2): e0228870 10.1371/journal.pone.0228870 32084161 PMC7034807

