# Supplementary material for: Performance under pressure in skill tasks: An analysis of professional darts
Source: PLoS One. 2020 Feb 21;15(2):e0228870. doi: 10.1371/journal.pone.0228870 (PMC7034807; doi:10.1371/journal.pone.0228870)
Supplement: Attachment — Submitted filename: Response_to_Reviewers.pdf [file pone.0228870.s002.pdf]

Point-by-point responses to the referees' comments on the revised version of our paper "Very Highly Skilled Individuals Do Not Choke Under Pressure: Evidence from Professional Darts"

We would like to thank all referees for their careful reading of our manuscript, and for their thoughtful comments and queries, which helped us to improve and strengthen the work. Following the suggestions made, we now present a very substantially revised empirical section with a new analysis approach. Briefly, the main changes we made in this revision are as follows:

- We do not categorize finishes as 1-, 2-, and 3-Dart-checkouts anymore, since the first referee suspected that not all scores within a given category are equally likely to be finished — we looked into this, and the data do in fact support the referee's suspicion. We addressed this issue by now considering as covariate information the overall checkout proportions for all individual scores considered. Any potential choking effect is then quantified as an additional shift in the checkout probability as a function of the opponent's chance to check out in his next turn.
- Related to the previous point, to conduct a more fine-grained analysis of performance, we present a further analysis based on single *throws* rather than on complete *turns* as done in the previous version of the manuscript.
- We now account for the intermediate score within a match. Specifically, we analyze subsamples where both players only need one more leg to win, since these situations decide between winning and losing — pressure should then be highest.
- We added references and discussion related to the literature on 'social facilitation' and 'clutch performance', which indicates that pressure can also enhance performance.

We also made a few smaller changes as requested, addressing all the comments that were made. Detailed point-by-point responses are given below, and all changes made in the paper are highlighted in yellow.

Response to Referee 1

Major Comments:
1. Wow, this is really great data! With that being said, the detailed data could allow for a more precise examination of performance than the 1,2,3 dart checkout situations. I agree with the analysis done and its result, but want to know more about the likelihood of checking out from certain scores. I am a little concerned that the 1, 2, 3 dart finish situations are too broad of a categorization for the difficulty to checkout. From my understanding of darts (playing and watching very little), along with the authors explanation of the rules and situation (great job there), maybe all 2-dart finish situations are not created equal, it could be that 56 is much easier to checkout from than 80. In addition to table 4, I would like to know the probability of checking out from each specific score at the start of a player's throw of 3 darts. A figure could easily display this information. Also, are there point totals from which a player's attempt to checkout happen more or less often? I would suspect there are common point totals which players start their checkout attempts from. To address this last point, the authors could add a histogram of the scores at the start of a players turn when they have a chance to check out. In general, my

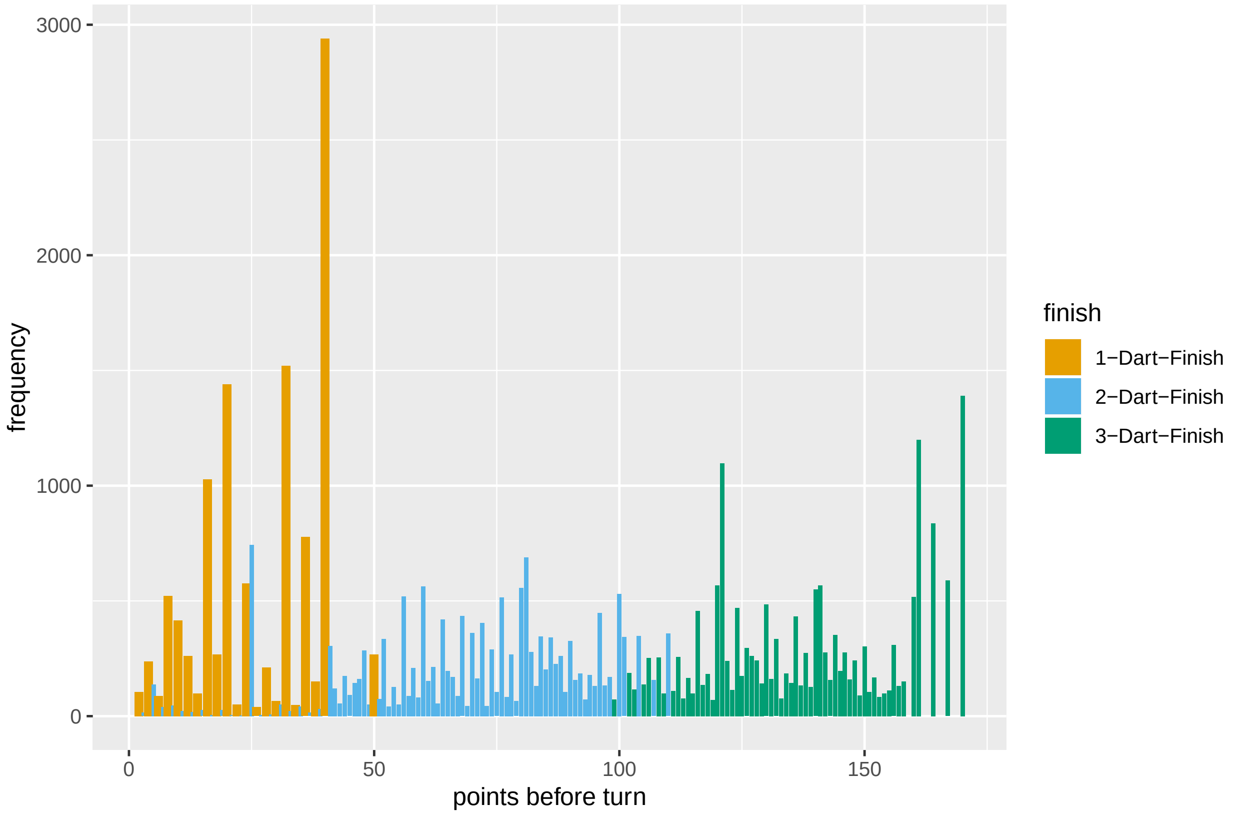

Figure 1: Empirical frequencies of scores (in the finish region) before a player's turn. Turns with both unsuccessful and successful checkouts are shown. Colors indicate whether the score corresponds to a 1-Dart, 2-Dart or 3-Dart-Finish.

> *concern is that there is some clumping of scores at a certain spot which make it much easier or harder for a player to checkout and the broad categorization used does not appropriately capture the difficulty.*

We are grateful for the referee raising this issue of the too broad categorization. We agree and have followed up on the remarks in the following way. First, we looked into the frequencies of scores before a player's turn, as shown in Figure 1 of this response letter. We find support for the referee's suspicion that players attempt to arrive at particular scores. Second, we verified that the checkout probabilities do indeed heavily depend on the score. This supports the criticism that the distinction between 1-, 2-, and 3-dart-checkouts is not fine-grained enough. Figure 2 in this response summarizes the empirical proportions.

In the analysis in the revised version of the paper we hence include the overall empirical proportions for a checkout for both players as additional covariates instead of considering the 1, 2, and 3 dart checkout categories (see Tables 2 and 3 in the manuscript). With this new measure of pressure and by considering strategic adjustments (see below), we find no effect of pressure on performance.

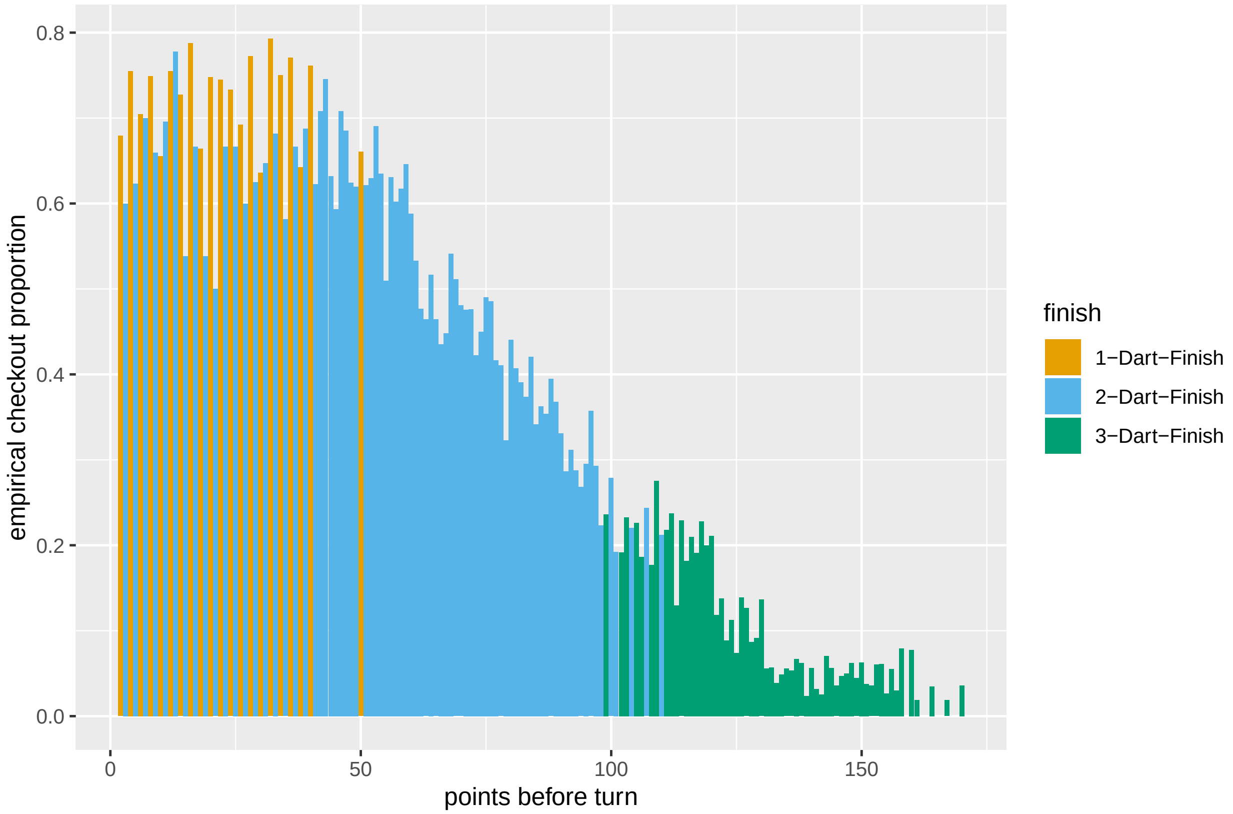

Figure 2: Empirical checkout proportions.

2. Another issue closely related to #1, is the mention of strategy in not closing out in footnote 6, this brings up some questions as it relates to the data and the strategy of not checking out. At the top of page 14, the description of the data mentions the researchers know "the score before each dart". All of the analysis in the paper is from the perspective of knowing the score before throwing all 3 darts when it is a player's turn. Footnote 6 gets more at this idea of having data on each single dart throw as it discusses excluded throws where "the player did not try to check out with his last dart". Since there is data on each dart throw, then why would you not look at specific dart throws where the player is on an even number and they need to throw the last dart of their turn into a specific double number? You still have the information on the probability for your opponent to check out, and you can more precisely measure performance in this moment of pressure. The 1,2,3 dart checkout scenarios could have many instances of strategies not to checkout when your opponent can't checkout on their next turn, or situations where there are mistakes made by the player which causes them to land on an odd number and have no ability to check out on their second or last dart.

These are again very useful suggestions. In the revised version of the manuscript, we present an additional analysis of the throwing performance based on single throws rather on all three darts of a player's turn. The results from this analysis suggest that the throwing performance is not affected by pressure (see Table 3 in the revised version of the manuscript and also further discussions on strategic adjustments below).

3. More specifically on footnote 6, it mentions situations when a player has a 1-dart finish, meaning that they have an even numbered score of 50 or less to start their turn. Well, they have 3 chances to make a double, and the footnote is correct that players often times may not go for the checkout

on the first dart and instead get their score to an easier position such that a double 8, 16, or 20 would give them a checkout. However, the example in the footnote only discusses two dart throws, when the player would actually have three darts throws in a turn, this makes the example a little confusing. Is the exclusion of 11 observations from the case when a player has a 1-dart finish and decides not to try and checkout on the last dart throw? First, I can see why there would only be 11 excluded in this case as it means that the player had to make a mistake on their two previous throws and then be on a number likely to bust or an odd number for them to decide to not checkout. The main point here, can't this same type of strategy happen on 2 and 3 dart finish turns? In general, strategy to not checkout may be driving the results in Table 5 as a 3-dart checkout is very difficult, players will often want to land on a good even number (e.g. 40, 32, 16) when their opponent can't checkout on the next turn.

Since our data do not indicate the segment players aim at, it is rather difficult to account for these strategic adjustments. However, in the revised version of the manuscript, we discuss the potential strategic adjustments in more detail. Figure 2 in the revised version of the manuscript compares the checkout proportions for situations where the opponent had a finish to those where the opponent had no finish. It indicates that especially for scores larger than 100, players rather tend to setup an easier score to checkout instead of checking out when the opponent has no finish. However, for scores below 50 (i.e. 1-Dart-Finishes), setting up a score is less relevant and the checkout proportions do not differ substantially between situations where the opponent had a finish and those where he had no finish.

We propose two different solutions to not mix failure and strategic behavior: First, we solely focus on situations where the opponent also has a finish. Second, as stated above, we consider an additional analysis based on single throws. Within this analysis, we focus only on the third throw of a player's turn and again on situations where the opponent has a finish to avoid strategic adjustments. We focus on third throws of a player, since the first two darts of a turn are often "marker darts" (see discussion on page 19 in the manuscript). By those taking strategic adjustments into account, we do not find any significant effect of pressure on performance anymore (see Tables 2 and 3 in the revised version of the manuscript).

4. In general, to keep the current analysis in the paper, there needs to be much more discussion of player strategy to checkout and convince the reader that not checking out is a failure in performance and not a strategy decision. A quick google search returns websites with discussions on strategies to closeout (e.g. http://www.sentex.net/ pmartin/patdarts/strategy.htm), meaning the objective can often be to setup an easier closeout next turn when you are in an advantageous position against an opponent.

As mentioned above in our response to the third comment we discuss potential strategic adjustments in more detail, and present Figure 2 to compare the checkout proportions of situations where the opponent has a finish to those where the opponent has no finish. In addition, we analyze subsets of the data where we can rule out strategic adjustments (i.e. subsets where both the player and the opponent have a finish). As stated above, the results of this analysis indicate that there is no significant effect of pressure on performance anymore.

5. Checking out is an integral part of the game, as mentioned in the paper, matches are won when a player wins somewhere between 7 and 15 legs. This means you will need to checkout at least 7 times in a match to win. I would think checking out in the first leg of a match would have a different level of pressure than checking out for a 7th leg win to take down the match. In this sense, the legs in a match have different levels of importance and pressure as the match progresses. This seems very similar to making putts early vs. late in a round or sinking three throws early in a basketball game vs. the final minute in a close game. Taking the analysis towards a comparison of checking out in pressure vs non-pressure would more closely mirror the situations in other sports research and allow for a result that could be easily compared to other studies. If this type of analysis led to a different result than golf or three throws, it would be very compelling.

Thank you for these suggestions. In the revised version of the manuscript, we include these suggestions by fitting a model to a subset of the data, where both players only need one more leg to win (so called decider legs). We again include the analysis of this subset in both regressions, i.e. for the models with the sampling unit being turns and single throws, respectively. Tables 2 and 3 displays the corresponding results, with the main result being that the finish of the opponent does not seem to affect the throwing performance in decider legs.

6. Section 5 which includes Tables 8 and 9 is a nice addition to tackle the issue of prize money. However, the discussion is incomplete, one sentence on the results from Table 9 in the last paragraph of section 5 is not nearly enough. Also, the discussion of expected results when adding these control variables states, "One would expect a higher Cb to be associated with lower incentives to perform well, hence a negative impact on the probability to check out". Table 9 results indicate a positive coefficient for the Cb variable, which I understand to mean higher Cb means more likely to checkout, the opposite of the pervious statement. Maybe I am misunderstanding this, but either way this needs some clarification.

Since a different referee suggested to skip Section 5 because there is no question that darts is a skill and not an effort task, we excluded this section from the manuscript. Still, we include the covariate Cb in our models to account for the heterogeneity between the players.

Minor Comments:

- *1. There are several typos, (e.g. 'in' instead of 'is', 'price' instead of 'prize').*
  We made an effort to remove all typos.
- *2. There is a recent paper that could be added to the discussion of golf and performance under pressure (Hickman, Kerr and Metz 2019). The study shows players perform better in 2nd vs 1st. This may fit the story in this paper of performing best in 2 and 3-dart checkout situations (slightly behind) when the opponent has a 1-dart checkout (slightly ahead) on their next turn.*
  We now refer to this paper in the revised paper.
- *3. My understanding of darts is that there are some nuances about having a number very close to zero and busting (going past zero) which likely deserves some discussion in the paper. There could be a situation where a 1-dart checkout where a single throw will either win you the leg or have you bust; this could be a throw with enormous pressure compared to other situations.*
  The remark suggests that a bust has a big impact on the players' performance. As a matter of fact the consequences are rather minor as the player can start with the exact number of point left as he had when he overshot and busted in the previous attempt. We refer to the term bust and the consequence of a bust in a footnote of the revised version of the paper.
- *4. While Table 7 is being used to try and indicate better performance under pressure, this result of all 122 players checking out more often when their opponent could checkout next turn vs. not checkout leads me to the strategy conclusion discussed in my previous comments. If all players are checking out less in the "no pressure" situation, then maybe the lower rate of checking out is a strategy decision for the end of leg play.*
  Table 7 is no longer part of the paper as our empirical approach is very different from the previous one. We now find at least some hints for strategic behavior in our paper. As part of the revision we show that not all players respond to pressure in the same way. Instead, some players improve while others decline under pressure. The corresponding analysis is presented in the appendix (see Figure 5 for descriptive statistics, and Tables 4 and 5 for the results of additional random effects for the performance under pressure).

Response to Referee 2

I was very interested in the manuscript. My reading suggested that the study was well-conducted and well-analyzed. The use of alternative models and alternative ways of approaching the data (including presenting the data from, an individual-difference perspective) is always a plus for a manuscript, in my opinion. My opinion is that the results are interesting enough to be publishable. However, I did not think that manuscript itself optimally packaged these findings.

> *1. One major source of my concern was rooted in my perception that the manuscript did not fairly reflect the "lay of the land" with respect to the possibility that performance can increase under pressure. For example, in attempting to describe when performance might increase under pressure, the authors make a broad distinction between effort tasks and skill tasks, and use that as a justification for the study (p. 2). The manuscript is written to imply that darts is more of an effort task, but that status seems be assumed. If my perception is correct, one concern is that the authors do not present any independent evidence validating that assumption.*

We see the point being made, but there is in fact no clear classification of darts as either a skill or an effort task. McEwan et al. (2013, "The effects of depleted self-control strength on skill-based task performance", Journal of Sport and Exercise Psychology) claim that throwing darts is a skill task as it is associated with a highly standardized movement which requires a high motor skill in order to perform well. We agree with this view even though we think the distinction between skill and effort task is not clear cut.

> *2. Moreover, the authors do not link to an especially relevant literature: social facilitation/social impairment. The fact that this literature is often overlooked by researchers who have focused on the absence of evidence for "clutch" performances (e.g., performance enhancement under pressure) has long mystified me, and given the ghosting of this literature, it is not surprising that the authors of this manuscript have also overlooked it. In my view, that is an important oversight. That social facilitation/social impairment literature is quite relevant to the issue treated in this manuscript. That literature suggests that arousal (which may be conceptually similar to "pressure") might improve performances on tasks that require the emission of "dominant responses." Roughly, these are responses that are well-learned in a given context. In fact, the demonstration of social facilitation effects has been confirmed by meta-analyses (see Bond and Titus, 1983), and has been linked to physiological, cognitive, affective, and self-presentational mechanisms. For examples, see: 1. Blascovich, J., Mendes, W. B., Hunter, S., & Salomon, K. (1999). Social facilitation as challenge and threat. Journal of Personality and Social Psychology, 77, 68-77. 2. Bond, C. F., & Titus, L. J. (1983). Social facilitation: A meta-analysis of 241 studies. Psychological Bulletin, 94, 265-292. 3. Harkins, S. G. (1987). Social loafing and social facilitation. Journal of Experimental Social Psychology, 23, 1-18. 4. Huguet, P., Galvaing, M. P., Monteil, J. M., & Dumas, F. (1999). Social presence effects in the Stroop task: Further evidence for an attentional view of social facilitation. Journal of Personality and Social Psychology, 77,1011-1025. This body of research is important to the present manuscript because it clearly points to three things: (a) task performance CAN improve under pressure; (b) the idea that whether one gets performance impairment or performance improvement may be moderated by the type of task to be performed, and (c) there are several kinds of psychological variables that may be linked to performance improvements. Indeed, I raise this literature because it contradicts the authors' assertion (on p. 2) that "However, there is broad agreement in the literature that performance in skill tasks, e.g. juggling a soccer ball (Ali, 2011), declines in high-pressure or decisive situations. "Prompted by my knowledge of the above literature, my immediate thought was "Oh yeah? WHICH literature?" It is certainly the case that, as is well-documented by the authors, both laboratory studies of sports-like performances and most analyses of real-world sports performances supports the conclusion that players are not "clutch" performers in that they do not improve performances under pressure. However, as I have noted above, there IS ALREADY A LITERATURE suggesting that for some kinds of tasks (maybe dart-throwing is one of them) and for some kinds of people (perhaps dart-throwing experts are those kinds of people), performances MAY improve under pressure (e.g., in*

high arousal states). One of my obvious recommendations, then, is that because it suggests that performance improvement under pressure may sometimes occur, the authors need to touch base with the social facilitation literature. However, it should be noted that other elements of the sports-like task performance literature has recently been more congenial to the performance improvement perspective than has, perhaps, characterized the past. Examples (not a comprehensive review) of this literature are:

Preventing motor skill failure through hemisphere-specific priming: Cases from choking under pressure. Beckmann, Jurgen; Gropel, Peter; Ehrlenspiel, Felix. Journal of Experimental Psychology: General. Vol.142(3), 2013, pp. 679-691. (see results of Experiment 3). Choking vs. clutch performance: A study of sport performance under pressure. Otten, Mark. Journal of Sport & Exercise Psychology. Vol.31(5), 2009, pp. 583-601.

Do clutch players win the game? Testing the validity of the clutch player's reputation in basketball. Solomonov, Yosef; Avugos, Simcha; Bar-Eli, Michael. Psychology of Sport and Exercise. Vol.16(Part 3), 2015, pp. 130-138.

Changes in putting kinematics associated with choking and excelling under pressure. Gray, Rob; Allsop, Jonathan; Williams, Sarah E. International Journal of Sport Psychology. Vol.44(4), 2013, pp. 387-407.

Pitching and clutch hitting in Major League Baseball: What 109 years of statistics reveal. Otten, Mark P; Barrett, Matthew E. Psychology of Sport and Exercise. Vol.14(4), 2013, pp. 531-537.

Choking and excelling at the free throw line. [References]. Worthy, Darrell A; Markman, Arthur B; Maddox, W. Todd. The International Journal of Creativity & Problem Solving. Vol.19(1), 2009, pp. 53-58.

Enhanced expectancies improve performance under pressure. [References]. McKay, Brad; Lewthwaite, Rebecca; Wulf, Gabriele. Frontiers in Psychology. Vol.3 2012, ArtID 8.

These citations support the notion that even in the "sports performance-like" literature, there have already been reports (especially in recent years) of increases in performance under pressure, as well as articulations of possible mechanisms by which such performances increase. I obviously would recommend that this manuscript touch base with this literature, and as a consequence, tone down (a little) the implication in their manuscript that their data are totally discrepant with the existing literature. This is especially important when the authors write: "Hence, following the literature, performance in darts would be expected to decline as pressure increases. Against the current state of research on motor tasks, we find (nearly all) professional darts players to excel in pressure situations. We argue that highly skilled individuals are able to digest pressure situations towards a positive outcome, while those who choke under pressure decide towards other professions. This emphasizes the importance of possession of skills for the impact of pressure on performance." As illustrated by the references I have provided the literature is NOT uniform in supporting choking under pressure. In this regard, then, I would recommend that the authors add a definition of the term "clutch" to their terminology section, that they review some of the studies suggesting that clutch performances can occur, that they link to some of the conditions under which clutch performances can occur, and that they describe some of the mechanisms that may produce clutch performances. This can all be done, of course, with the caveat that in the literature choking performances tend to be much better documented and seem to be more common than clutch performances. Note that, in my view, my "packaging concern" does not diminish the interest value of the authors' findings. Only via the publication of these kinds of data will one gain insight into (what I believe) are the critical questions: (1) on what kinds of tasks will performance increase under pressure and on what kinds of tasks will it decrease, and (2) what are important psychological factors that are at play in such changes (e.g., expertise, attention, etc.). The authors seem to define choking under pressure as "specifically to a negative impact of high performance expectations (Baumeister and Showers, 1986; Hill et al., 2009)." That definition may be a little

restrictive. Choking may also be viewed through a motivational lens in that it may require the desire to do one's best.

This is a great point — 'social facilitation' does indeed provide highly relevant explanations for our findings as our empirical results do not confirm choking. We very much appreciate the referee pointing us towards this field of research. As stated by the referee, studies on choking under pressure typically do not refer to this literature. This is most likely why we (also) overlooked it.

The social facilitation literature provides valuable insights and helps us to explain our findings. We have thus added literature on social facilitation to our theoretical section (p. 5f) and explain Zajonc's (1965) assumption that pressure facilitates dominant behavior and hence, may — under certain circumstances — enhance performance. However, we also describe that social facilitation research provides a wide variability with respect to causes and consequences of such behavior.

We further added several empirical studies covering clutch performance to our section on previous findings (p. 6-10). Accordingly, we also refer to social facilitation in the introduction and argue that theoretical and empirical literature exist confirming both negative or positive effects of pressure (p. 2f). In the revised version of the paper we leave open whether pressure should affect performance of dart players in a positive or negative way. Overall we move away from a rather one-sided (negative) presentation towards a more universal consideration of potential pressure effects. The term 'clutch performance' is explained in Section 2.1 of the revised paper (p. 4).

3. I understand that section 2.4 is intended to try to provide a context for why dart throwing might be a particularly good context to investigate how performances might change under pressure. I agree that the sport of dart throwing has advantages (relatively standardized conditions, outcomes that are not influenced by opponent) that might serve to quiet random variance. However, a counterargument lies in convergent validity: when an outcome (e.g., choking under pressure) is repeatedly observed across very different contexts, one gains extra confidence in the outcome because the confounding variables that are unique to a given context are often not present in other contests. Hence, with convergence it is increasingly unlikely that variables unique to a context caused the result. Thus, although soccer penalty kick outcomes are directly influenced an opponent, golf tournament performances are not. Similarly, while laboratory studies of choking often use non-expert participants, field studies often show the same choking phenomenon in experts. The authors imply that dart thrower expertise is crucial to the clutch performance data that they report (e.g., "The stark difference between our findings and previous studies may partly be due to the fact that in our study we consider very highly skilled individuals who have to deal with 21 the considered type of pressure situations on a regular basis."). Given results from other field research employing experts, this seems to be an unlikely explanation. To be more specific, as implied by the social facilitation literature, expertise is NOT the only variable that may be important to clutch performances. Studies of many sports (e.g., golf; baseball) have shown that even expert players sometimes choke, or they sometimes do not exhibit either choking or clutch in their performances.

As the revised empirical analysis shows findings that are quite different from those presented in the original paper, our discussion has changed accordingly (see p. 22ff). However, we still relate our findings to the fact that the darts players observed in our sample are highly skilled individuals since expertise is, as stated by the referee, a relevant aspect in the social facilitation literature. We do however broaden our interpretation towards further aspects such as self-confidence.

4. I had concerns about the notion of "pressure" derived from Table 1 and Table 2. I understand that Table 1 is presented to provide a basis for the pressure labels presented in Table 2. However, there is no justification for the Table 2 categorical labels applied to the conjoint probabilities (no, low, moderate, high) depicted in Table 1. The authors' labels suggest that their view of pressure is determined by how easy both the player's task is (making one dart is easier than making three darts) and how easy the opponent's task is. However, this system is not well articulated in the

manuscript, and no external validation evidence for the system is presented. Extrapolating from psychophysics research, one needs external validation to determine how much felt pressure changes as the win probability changes, and whether and how felt pressure is related to both the difficulty of one's own task as well as the difficulty of the opponent's task. Might it be the case that the two variables do not contribute equally to felt pressure? For example, I can easily see that my task (1 vs. 2 vs. 3 darts needed to win) might contribute more to felt pressure than my knowledge of my opponent's task. Moreover, why must these probabilities be translated into categories? Could they not be used as a continuous predictor as a proxy indicator of pressure In addition, one cannot always expect the change in felt pressure to be linearly related to the change in probability across the probability scale. That is, a change in win probability from.95 to .85 may not produce the same change in felt pressure as a change in win probability from .15 to .05. I don't know if these worries are enough to derail the manuscript, but I certainly do think that a thorough discussion of links between pressure and win probability, and the assumptions that the authors made in translating win probability data into pressure categories, is needed.

The fully revised empirical section aims at a clearer identification strategy compared to the manuscript initially submitted. (High) Pressure situations are now identified by observing throws which are not only responsible for winning a lag, but for winning the game. We do not have to rely on self-defined levels of pressure on the players (formerly defined categorically as 1, 2, and 3 dart checkout situations), but can now assure to separate the most important situations from the rest of the game to derive the impact of pressure. The new estimations show that performance in high pressure situations is highly comparable to performance in situations where the pressure is considerably lower. Accordingly, we find no impact of pressure on performance.

One aspect that you mention cannot be captured by our paper, namely to identify the pressure felt by a player. We have to assume that the pressure on a player is determined by the score at the time of the throw and other control variables we refer to.

5. Given where it is placed, the inclusion of ideas from contest theory (p. 20) seems like an afterthought. If contest theory makes a priori predictions about the patters pf data to be expected, those predictions seem to be better placed in the introduction near to where the effort vs. skill distinction is first raised. Note that this effort vs. skill distinction is not the same as presented in the social facilitation/social inhibition literature. The literature comparable to the "effort" literature would probably lie in the social loafing domain of social psychology. However, I wonder about the relevance of the effort idea. Effort has not generally been posited as an explanation for choking (given the assumption that people choke, even when they are trying to do the best that they can). Hence, I doubted whether this effort-related material added much to the manuscript.

We agree with the referee. The revised version of the paper offers new modeling approaches and we defer from including a variable covering prize money. Our theoretical considerations now do not cover ideas from tournament theory any more.

6. One other very minor point is that I do not know if Frontiers requires articles to be formatted in compliance with the guidelines set forth in the APA publication manual. If that is the cased, then this manuscript exhibits frequent noncompliance. A partial list of noncompliant items includes missing page headers, missing figure captions, misformatting table captions, and the misformatting of references (e.g., issue numbers are no reported only in special cases; no colons before page numbers; use the ampersand instead of the word "and"). If compliance to APA style is required, the authors clearly need to sit down with the manual and alter their manuscript to comply with the manual's guidelines.

In the revised version of our manuscript, we now use the Latex template provided by PLOS ONE. In addition, we added captions for all figures and tables, which are also listed in the supporting information at the end of the document. We made an effort to provide complete references by adding all missing issue and volume numbers as well as page ranges.

Response to Referee 3

I had two primary concerns with this paper, both of which are related to how choking under pressure in conceptualized in this context:
1. As noted in my summary above, the authors defined a high-pressure situation as one in which the win probability was lower (because the opponent was doing better), conditional on having a shot to win the leg, or checkout, that turn. However, I am concerned about the aptness of this definition, on which the paper's conclusion of course relies. While it is difficult to provide an objective definition for a "high-pressure" situation bar, e.g., capturing biometric measures of stress, I would not think that pressure would be increasing in the opponent player's chance of winning, conditional on having the opportunity to check out yourself. Consider, for instance, a case where a player has a 50% chance of winning compared to a case where a player has a 1% chance of winning; I would say that the first case is higher-pressure because "there's more on the line to lose" (this could be framed in the context of expectations-based reference dependence, where the loss in utility following a lost opportunity to checkout is not as substantial when a player has a 1% chance of winning because their reference point was that they would fail to do so). In fact, defining a high-pressure situation as one in which a game is "close" is an established approach in the literature (Cao et al, 2011; Toma, 2017). Yet this is different from how the authors define choking in this paper, where the lower the chance a player has of winning, conditional on having a chance that turn, the higher the pressure, even when this means that the game is actually less "close" as in the example above. Since the evidence of choking under pressure presented here is of course contingent on the definition of a high-pressure situation, this naturally substantially affects my perspective on how the results ought to be interpreted.

We agree that it is somewhat difficult to objectively define high-pressure situations, and thus have generally toned down the interpretation of our results accordingly. In the revised version of the manuscript, we further present an additional analysis focusing specifically on close matches. For that purpose, we analyse a subsample of the data in which both players need only one more leg to win the match (so-called decider legs). The throwing performance within these situations is illustrated in Figure 4 in the manuscript. In only about half of the finishes the checkout proportion is higher in decider legs, indicating no clear pattern. We conducted the analysis of this subsample both at the level of a turn and at single-throw level (the latter is new in the revised paper) — see Tables 2 and 3 in the manuscript. In addition, based on the comments by Referee 1, we now do not distinguish between 1, 2, and 3 dart finishes anymore, since this categorization is too coarse (see response to point 1 of Referee 1 above).

2. A crucial assumption the authors must make in order to identify choking under pressure in this context is that all else is held constant when comparing play across situations in which the opponent is one dart, two darts, three darts, or more away from checking out. However, my understanding is that players may adjust their strategy according to their opponent's position. For instance, if their opponent has no chance of checking out during their next turn, a player, especially one who needs either two or three throws to finish, might play more conservatively, throwing in a way that somewhat decreases their chance of checking out this round but positions them better for the following turn. This interpretation is exactly consistent with the results: When a player needs either two or three throws and their opponent cannot checkout their next turn, they are significantly less likely to checkout on the turn in question compared to a case where it is possible for their opponent to checkout; this difference is no longer significant when the player needs just one dart to checkout and thus conceivably has a more narrow strategy set. Thus, the higher probability of checking out on a given turn when the opponent has the chance to check out on their next turn seems quite conceivably due to the differences in optimal strategy across these cases rather than improved performance under pressure.

The ideal analysis would control for these differential strategies across cases (and this is why free throw or soccer penalty kicks are an appealing context in which to study choking – there is just one optimal strategy regardless of the position in the game: try to make the shot/goal). One possible

approach would be to compare performance under these different scenarios (the darts required for self vs opponent) when the overall match is close vs not. If we observed that players were more likely to checkout when the overall match was close, conditional on the position they were in in a given lag, this would seem to provide more convincing evidence that darts players indeed excel under pressure. Footnote 7 suggests that no significant results were found using this analysis (which I would think should be highlighted!), though it was unclear whether this specific test was employed.

Based on these suggestions, we now compare the empirical checkout proportions for situations where the opponent has a finish to those where the opponent has no finish (see Figure 2 of the revised manuscript). The figure does indeed suggest that especially for remaining scores larger than 100, players tend to set up an easier checkout instead of checking out in cases when the opponent has no finish. With a view on this strategic behavior, we now fitted both the turn-level and the throw-level models to only a subsample of the data where *both* players had a finish, since in these situations there is only one strategy, namely going straight for a checkout. Moreover, when analyzing single throws, we only consider the third throw within a turn to avoid further strategic adjustments of so-called "marker darts" (see discussion on page 19 in the manuscript). We do not find any significant effect of pressure when restricting the sample to observations where both players had a finish (see Tables 2 and 3 in the manuscript).

Regarding the second comment on close matches, as already stated above, we compare decider legs to non-decider legs and find no effect of pressure on performance in both situations.

In addition to these two main concerns, noted below are strengths of the paper as well as some additional concerns and suggestions:
1. I appreciate that the authors took seriously the psychology literature that differentiates, for instance, the theoretical predictions of the impact of choking in effort- vs skill-driven tasks – this is an important literature to understand when exploring performance under pressure and the authors did this justice in the literature review. One suggestion, however, would be to avoid generalizing the findings in this paper to behavior in the labor market in the abstract and conclusion, as this is exactly an instance of crossing from a skill-driven to a (typically in the labor market) effort-driven task, and, as noted, the theory of choking under pressure would predict very different types of behavior across these domains.

We appreciate and agree with the referee's argument. Thus, in the revised version of the paper we have toned down the link between the outcomes of our paper and the labor market.

2. The paper includes a section on why other studies may not have correctly identified choking under pressure. I indeed believe that this is an important section to include in order to credibly assert that the novel findings in this paper may be true across the board, but I had concerns about a number of the claims made:

a. *The authors argued that it's difficult to disentangle pressure from "nuisance situations" such as weather conditions or the quality of the course. However, I see no reason to believe such nuisance conditions would be correlated with high-pressure situations, and so this should not pose a challenge to the identification of choking under pressure. Similarly, they argue that the small number of e.g. penalty kicks in soccer render the data sufficiently noisy as to make choking difficult to tease apart — I would see this as a specific concern if player fixed effects cannot be netted out (as in Dohmen, 2008) such that you may just have stronger players kicking in high-pressure situations, but otherwise a small number of observations alone should not invalidate results that have been found to be significant.*
   We are grateful for these very useful thoughts. In the revised version of our manuscript, this section has been rewritten in response to the referee's issues a–d as cited here, now emphasizing the advantages of the data at hand. Specifically, we completely agree with

the referee's arguments concerning the "nuisance situations" and do not state anymore that it is difficult to disentangle pressure from "nuisance situations". Moreover, we now argue that the large number of observations in darts are beneficial, since estimating skill levels in pressure situations requires a comprehensive data set. With hundreds of throws for each player contained in our data, we can derive a relatively stable baseline performance and disentangle the impact of pressure.

b. *The authors note that fatigue may enter in as high-pressure situations are those in which players are more likely to be fatigued because they have been playing longer or in a more intense game. Toma (2017), however, addresses this using free-throw data by showing (1) that choking only occurs in the final 30 seconds of a close basketball game and not before, when players are presumably similarly fatigued, and (2) that the number of seconds a player has played in a game does not predict choking.*

   Since we now discuss advantages of darts, we argue that fatigue does hardly impact performance in throwing darts. At the same time, we state that this may be different in other settings, while we also explicitly note that Toma (2017) shows fatigue not to impact performance in by analyzing free-throws in basketball.

c. *The authors argue that coaches may tend to put players best able to handle pressure in high-pressure situations, but this would seemingly work against the finding that players choke under pressure.*

   In the revised version, we have weakened our claims regarding the decision of coaches to consider players who do not choke under pressure. Specifically, we argue that players in darts are confronted with high pressure on a regular basis and cannot get away from those.

d. *The remaining points are specific only to certain types of analyses (e.g. soccer or experimental data) and thus cannot explain why findings across the board may not be valid.*

   We reduced and rephrased the perhaps too critical remarks regarding the existing literature.

3. These data seem like an interesting opportunity to look at heterogeneities in choking across players (once the right definition of choking is nailed down!), and while I appreciate that the authors took a step in this direction I think there's more that could be done with the analysis:

a. *First, just a somewhat picky note that the authors' claim is not seemingly supported by their evidence given that they do not have any data suggesting others may choke under pressure: "We argue that highly skilled individuals are able to digest pressure situations towards a positive outcome, while those who choke under pressure decide towards other professions."*

   We rephrased the respective argument. It is correct that we cannot show that others indeed choke under pressure or decide for other professions.

b. *I like the idea behind Figure 2, but I think I would have found more informative a graph with actual rather than predicted checkout probabilities across opponent scores for players with sufficient data; that way, not only the intercept would change, but we would actually see differences in choking susceptibility (this is seemingly a motive underlying Model 2, which I appreciate!). Perhaps in a new version of Figure 2 bins of different player "types," by choking propensity, could be created and graphed.*

   For the subsample mentioned above where we can rule out strategic adjustments, i.e. situations where the opponent also has a finish, we compare the empirical checkout proportions for different situations of pressure in Figure 3. Specifically, we compare situations where a checkout of the opponent is rather unlikely (≤ 0.3), those where the checkout proportion is between 0.3 and 0.6, and situations with checkout proportion of the opponent larger than 0.6. For scores below 50 (i.e. 1-Dart finishes), the checkout proportions seem to increase the more likely the checkout of the opponent gets, whereas for higher scores there is no such clear trend.

In addition, to also consider heterogeneity between players, we compare the checkout proportions for the third dart of a turn for situations where both players have an "easy" finish (implying the scores 2, 8, 16, 22, 32 and 36, which have a checkout proportion of at least 0.4) with those where the opponent does not have an "easy" finish. The corresponding analysis is presented in the appendix. Figure 5 shows the corresponding proportions. While we find some differences between the players, including random effects for the performance under pressure in the model formulation does not improve the model fit and the overall effect remains statistically insignificant (see Table 4). Table 5 in the manuscript summarizes the corresponding player-specific effects of pressure on performance, indicating that the effect may be positive for some players, whereas for other players the effect is negative.

4. The authors work with a great, fine-grained dataset, which is exactly what would be required for this type of analysis!

In the revised version of our manuscript, we made an effort to exploit the fine-grained darts data at hand. Specifically, we made use of the information on each *throw* and fitted a model to this data. We also provide an analysis of decider legs (and hence of the intermediate score) for this type of data. As mentioned above, our data contain several observations per player, which allows us to investigate heterogeneity between players (descriptive statistics shown in Figure 5 and a corresponding fitted model presented in Tables 4 and 5).

Some minor additional comments:
1. It took some time for the difference between Table 1 and Table 4 to sink in for me – My interpretation of the difference is that Table 1 looks at the probability of winning the leg overall while Table 4 looks at the probability of checking out that turn. This would be helpful to clarify, especially as the logic behind why the two are differentially relevant for the analysis was not made clear (this gets back to my second point -– presumably the probability of winning a leg can't be considered as a main outcome because this is dependent on opponent play, but even the strategy for the current turn is influenced by the opponent's standing, so this still seems like a concern when considering checking out for a given turn).

Due to the changes requested by the referees, these tables are no longer part of the paper. Strategic behavior is now investigated in more detail (cf. Figure 2). Since our data suggests that there is indeed some strategic behavior, we fitted our models to data where *both* players had a finish, to not mix up strategic behavior and performance under pressure (see also the response to the second point of the major comments). In addition, all figures are now also based on the corresponding subsample where both players had a finish (see response to the next point).

2. For more visually-oriented folks, it may be helpful to plot the degree to which the changes in probabilities of checkouts correspond with the defined pressure in the situation; in other words, create a figure combining the information in Table 1 and Table 2 such that pressure is on the x axis and the probability of checking out is on the y axis.

Although these tables are no longer contained in the manuscript, we offer some additional figures to investigate the effect of pressure on throwing performance. As our new empirical approach is now much more fine-grained, we believe our revised paper also serves substantially better vizualisation.

The empirical checkout proportions are shown in Figure 3, where colors indicate different finish-categories (and hence a different degree of pressure) of the opponent's finish (see also the response to point 3b). For pressure situations as indicated by decider legs, Figure 4 shows the empirical checkout proportions, indicating that the throwing performance is not substantially different in decider legs compared to non-decider legs. Finally, to investigate heterogeneity between players, Figure 5 presents checkout proportions in pressure situations vs. non-pressure situations for several players, indicating that ther are some differences between players (see again the response to point 3b). To account for the strategic behavior discussed above, all figures shown in the manuscript are based on situations where both players had a finish.

3. I think there is no question that darts is a skill- rather than effort-driven task, so I did not find the discussion in Section 5 to be necessary. That said, some of the variables included in this analysis, e.g. the prize amount, would seemingly be strong proxies for the pressure in a situation (the higher the prize amount, the higher the pressure) and so could be incorporated in this way. The null effects of the controls alone suggest to me that, consistent with the null effects in the choking literature often found for e.g. more important tournament games, players may internalize more the pressure of making vs breaking a play at a given moment rather than any larger stakes of an overall game that wouldn't be localized to a particular moment.

While we are convinced that throwing darts requires skill and not effort, we believe it is important to include some discussion on the topic into our paper. That being said a different referee even requested a stronger clarification than the one we had in the initial submission. We try to keep the balance as we add studies which refer to darts as a game of skill without deepening the discussion itself.

4. The statistical analysis tests for significant differences in checking out across opponent position only compared to the reference of the opponent not having the opportunity to check out the next turn; it would be useful to test for significant differences across the different opponent positions as well.

The statistical analysis is now a different one compared to the original submission since we do not categorize the finishes in 1, 2, and 3 dart finishes anymore. While we agree with the point made, it is not relevant with respect to the revised paper.
