# Supplementary material for: Performance under pressure in skill tasks: An analysis of professional darts
Source: PLoS One. 2020 Feb 21;15(2):e0228870. doi: 10.1371/journal.pone.0228870 (PMC7034807; doi:10.1371/journal.pone.0228870)
Supplement: Attachment — Submitted filename: Response_to_reviewers.pdf [file pone.0228870.s003.pdf]

Point-by-point responses to the referees' comments on the revised version of our paper "Performance under pressure in skill tasks: An analysis of professional darts"

We again would like to thank all referees for their careful reading of our revised manuscript. We made an effort to address all comments that were made. The additional analyses as motivated by the reviewers' comments we believe have improved the level of persuasiveness of our paper, while comments concerning the interpretation of our results have improved the readability.

We also made a number of smaller changes as requested. Detailed point-by-point responses are given below, and all changes made in the paper are highlighted in yellow.

Response to Referee 1

> *1. The figures in the revised paper are quite cluttered and it is difficult to see exactly what is going on with so many data points and lines. Page 9 of the revision states "the more points are needed, the less likely is a checkout (see Figure 2)." The second figure in the response to reviewers is a viewer friendly manner in which to display this information than the current Figure 2 in the revised paper. While the revision removes analysis using the number of darts to finish, I think a discussion of the 1,2, and 3 dart finish could be helped by using the second figure in the response to reviewers. Along this same general thought, the first figure from the response to reviewers does an excellent job of showing there are a handful of very common point totals from which players start their turn with an opportunity to check out. The first figure in the response to reviewers could be included to help the discussion of strategy and common starting points on pages 7 and 8 of the revision. What if the figures you currently have in the revised paper (Figures 2 thru 4) only displayed point totals to checkout with more than 500 or 1,000 observations? This could help with clarity as the figures are currently quite messy with all the points. This limitation would leave anywhere from 10 to 20 data points, making it visually easier for the reader.*

In the revised version of the paper, we made the following changes based on the referee's useful comments above: First, we have included the second figure from our previous response letter in the actual paper (cf. the new Figure 2 in the revised paper). The colors help to visually identify the differentiation between 1, 2 and 3 dart finishes. Second, we followed the advice to update Figures 3–5, which now include only those checkouts with at least 100 observations. As expected by the referee, this restriction of checkout numbers with low numbers of observation led to much tidier and hence easier-to-read figures.

> *2. As mentioned in the response to reviewers, the first figure with the frequency of different point totals to checkout would seemingly indicate certain point totals for 1-dart finishes are extremely popular and thus there is likely a strategic element to start the next turn with those exact points. The authors take this to mean, lets exclude all the checkout attempts when the opponent can't checkout on their next turn from the regression. As I might argue, strategy doesn't exist when we have a 1 dart finish starting at a popular number. For example, 40, which would be double 20 to checkout, has an enormous amount of checkout opportunities. Could you not look at how players perform when an opponent has chance to checkout next turn vs. not at this common number? This information is buried there in Figure 2, but I think there is still an argument to include the inability of the opponent to check out as a part of the regression. What needs to be made*

sure of is that the player is not choosing to strategically avoid going for the check out. To me, an opponent with a chance to closeout when I am at 40 would represent pressure and opponent without a chance to closeout would be no pressure. Of course, there are varying probabilities for your opponent to checkout, and your regressions are capturing how this may impact the player, but you are also removing many instances when the player is unlikely to be making a strategy choice to not checkout. This comment is a very lengthy way to say, restrict the sample to 1-dart finish situations for the player and report those results as well, but include in the regression throws when the opponent cant checkout on the next turn as well.

That again is a valid point. Table 1 below shows the results when restricting the sample to 1-dart finishes. Here, the dummy variable *OppFin* indicates if the chance to check out is impacted by the possibility of the opponent to check out during his next turn. The results show no impact of the variable *OppFin*, which again indicates that performance is unaffected by pressure. Given that we already have quite a few tables in the paper, we decided to restrict the presentation of these additional results to a remark in Footnote 8.

Table 1: Estimation results for the fixed effects when replacing the *CheckoutProportionOpp* covariate by the dummy *OppFin*.

	Dependent variable:
	Checkout
CheckoutProportion	4.826***
	(0.588)
OppFin	0.009
	(0.083)
Exp	0.002
	(0.004)
Cb	−0.009
	(0.115)
Constant	−2.576***
	(0.453)
Observations	10,075
Note:	*p<0.1; **p<0.05; ***p<0.01

3. The "pressure" variable in the revision is the probability of your opponent checking out, but doesn't pressure depend on a player's chance to win or lose the leg, the difference in probability to checkout between the players. As the analysis is currently setup, one interprets the CheckoutProportionOpp as the impact the opponent's chance to check out has on the probability of the player checking out, holding constant the players chance to checkout. This is possibly quite different from a story where the leg is a close competition and I feel pressure, the current "pressure" variable in the revision could include many scenarios when the leg is not a close competition. I suggest using the difference in checkout probability as a measure of pressure, with the argument that greater pressure exists when the difference is very small and less pressure exists when the difference is large.

Considering the difference in checkout proportions would not generally reflect whether the competition in a leg is close or not. For example, if both players have a remaining score of around 160, corresponding to a checkout proportion of 8%, then the difference in the checkout proportions would be ~ 0, however the leg should in fact not be considered as a close competition: due to the small checkout proportion of 8%, if a player does not checkout 160 points, he knows that it is fairly unlikely that his opponent will, and in the subsequent turn he will himself have a very good chance to checkout. If, on the other hand, both players have a checkout proportion of about 75% (corresponding to, e.g., a score of 40), the leg would be fairly close: in that case, players know that the opponent is likely going to checkout in his next turn, and hence feel more pressure. However, the difference in that case would again be 0. Hence, by considering the difference only, close situations in legs could not be clearly identified.

> *4. Table 1 in the revision indicates that CheckoutProportion ranges from 0.027 to 1, the second figure in the response to reviewers indicates the probability to checkout from any single number to be at a maximum of approximately 0.8. Is this difference a mistake or is there a part of the sample restriction that makes the probability of 1 occur? You could remove observations where the likelihood of checking out is extremely high (e.g. 1) or extremely low.*

This was indeed a mistake in the previous response letter. We restricted the sample to observations where both players had a finish, but the figure was generated without this restriction. The corrected figure is now also included in the manuscript (see Figure 2). We are grateful to the referee for spotting this.

Response to Referee 2

> *1. This is my second viewing of this manuscript. The authors received a considerable amount of feedback on their first draft, and they obviously worked very hard to incorporate that feedback into their revision. The authors' responsiveness is to be commended. More importantly, in my view the authors have produced a new manuscript that, in my opinion, is better than the first.*
>
> *It is good enough to rise above the publication threshold? My reading of this document prompted me to have some concerns that would give me some hesitation about seeing this version published. One concern that I had was expository. While making a substantial effort to incorporate reviewer feedback into their new draft, the authors never quite seemed to be able to abandon some of the approaches now conflict with the new revisions. Thus, the manuscript was not presented in a manner that was as coherent and internally consistent as one might like. If a revision is again called for, I would ask the authors to go through their document carefully to ensure consistency across the document.*

We appreciate the feedback on the effort invested in the previous revision. Given the very substantial revision in the previous round, there may indeed have been some inconsistencies in the presentation due to conflicts between renewed and old parts of the text. In this second revision, we made an effort to rectify these problems, in particular following the referee's further remarks as quoted below.

> *2. For example, in the current draft the authors continue to expound on the "advantages" of the darts setting. This includes that (1) performance is not directly influenced by others, (2) performers are highly trained; (3) task to be performed in a pressure situation is more or less identical to the only task the players perform throughout the contest; and (4) all players in darts are repeatedly confronted with high-pressure situations. In what way(s) are these advantages? One way in which these are an advantage may be statistical: The relatively individualistic nature of the task may work to quell stray variance in analyses (e.g., caused by the interference form the performances of others). However, the rest of the features? These characteristics are certainly not unique to darts: Many such as bowling would seem to present at least one of the characteristics. Indeed, many sports (e.g., bowling) may present them all. Moreover, the fact that darts players are highly trained, while true, is relatively meaningless for those tests of choking that have found evidence of choking in tasks where everyone in a highly trained expert (e.g., top-level professional sports).*

We agree with the points being made here. Our main aim is to point out potential problems of previous studies on performance under pressure — darts does have several advantages in that respect, but it is indeed true that other sports seem equally well suited. Overall, we strongly believe that darts constitutes a very good setting to separate the impact of pressure from other causes. The revised paper clarifies these arguments.

> *3. Moreover, I do not think that in making this claim the authors have quite abstracted one lesson of the social facilitation/social impairment literature: that the characteristics of the task help to determine the outcome one sees in co-actor performance situations (which presumably are one form of pressure situations). Hence, the nature of the task that is performed will help to determine whether one observes clutchness, choking, or nothing at all in performances. Hence, from the social facilitation/social inhibition view, the things that the authors list are not "advantages" but instead are "task features" that may help to determine the outcome that one observes. Indeed, one of these features (the fact that the motor skill involved (dart throwing) is relatively simple in comparison to many other motor skills needed in sport) would lead me to the prediction that in darts tasks the phenomenon of choking under pressure should be reduced, eliminated, or even reversed (e.g., clutch). It is this point that, for me, is where the manuscript has its interest. The "choking" literature has focused on choking, but the social facilitation/social impairment literature suggests that choking in pro sports may not be inevitable. Some circumstances may produce evidence of "clutchness," and some might produce no difference from non-clutch situations. The task is one of the controlling variables, and the facilitation/impairment literature says that the more the task relies on simple, well-rehearsed responses, the smaller the chance of performance decreases (choking).*
>
> *I would like to see the authors more explicitly and forcefully advocate this position. Why? The authors mention in their cover letter that they did not know about the social facilitation/social impairment stuff because the choking literature never cites it. Thus, in my view the authors can educate the choking researchers about the lessons learned about performance and pressure that have already been documented.*

We thank the referee for these insights, which led us to revise the explanations with regard to the social facilitation literature. The old version of our paper was indeed essentially a "choking" study amended with social facilitation aspects. In the new version, we advocate a more neutral position with respect to both positions (choking vs. social facilitation), and present the merits of the social facilitation literature more prominently. We have added several passages and subsections on relevant aspects of social facilitation with respect to our analysis (Introduction, Potential Effects, Task Features), so that we hope to now make sufficiently clear that our findings and results from the existing choking literature may be due to characteristics of tasks and individuals.

> *4. In this regard, the authors need to clean up their language a bit. At one point, they describe social facilitation as a "theory." That's wrong. Social facilitation/social impairments are observed performance outcomes. These outcomes supposedly reflect the impact of various arousal-prompted mechanisms (mere presence, evaluation apprehension, cognitive distraction) on tasks, with the outcomes theoretically moderated by individual expertise and task simplicity/difficulty (that's the theory).*

Again and in accordance to the previous remark, we revised our wording as well as our explanation on social facilitation / social impairment and the connection to the task performed in our setting. We appreciate these useful suggestions.

> *5. The authors probably over interpret their data. Sure, on a purely descriptive level, some results seem to reflect clutchness, and other results seem to reflect choking. However, in reality, in the authors' analyses none of these effects is significant. Hence, unless results are so strong that they are "trending," in my opinion it is probably best practice to simply characterize all the results as non-significant (and some would even use this characterization for "trending" results.*

In this regard, stats mavens like me would probably like to know the p-levels of the non-significant choke/clutch tests presented in Tables 2 and 3.

We agree with this sentiment. We revised the manuscript with respect to the usage of the terms clutchness and choking within our empirical analysis. Regarding the second comment, please find Tables 2 and 3 below (which have also been updated in the manuscript), now with p-values reported in every third line below the respective standard errors.

Table 2: Estimation results for the fixed effects of the turn-level model.

	Response variable:		
	checkout		
	(1)	(2)	(3)
CheckoutProportion	5.132***	5.128***	5.715***
	(0.058)***	(0.058)***	(0.536)***
	p = 0.000	p = 0.000	p = 0.000
CheckoutProportionOpp	0.014	0.016	−0.108
	(0.053)	(0.054)	(0.423)
	p = 0.798	p = 0.766	p = 0.799
Exp	0.005*	0.005*	0.007
	(0.003)*	(0.003)*	(0.018)
	p = 0.099	p = 0.090	p = 0.697
Cb	−0.018	−0.014	0.107
	(0.068)	(0.068)	(0.545)
	p = 0.785	p = 0.842	p = 0.845
Constant	−2.799***	−2.797***	−3.084***
	(0.062)***	(0.062)***	(0.456)***
	p = 0.000	p = 0.000	p = 0.000
Observations	32,274	31,715	559

Note: *p<0.1; **p<0.05; ***p<0.01

6. Moreover, I might argue that tests of significant effects have not provided the strongest tests of clutchness/choking. Might not this optimally strong test involve an analysis of the CHANGE in the effect obtained on low-pressure vs. high-pressure situations? In this regard, then, I might have expected to see whether performance changed significantly for the checkout proportion opp variable from the no deciders trials to the deciders trials.

This is a nice suggestion. Table 4 below reports the corresponding results, now covering both decider and non-decider legs and including the dummy variable *Decider*, which equals 1 for decider legs. As the variable in insignificant, this is another hint that the players' performance is not impacted by high-pressure situations. We were unsure if that table should make it to the main document, at it stands it is only part of this response letter while we mention the findings in the revised paper. As these results are in line with all other results, we decided not to include the table in the main manuscript. However, we do mention these additional analyses in the text on page 13.

7. Though non-significant, the fact that the data pattern may have shifted from the no decider trials to the decider trials (an analysis new to this version) again illustrates an important point that I made in my earlier review – that one needs to be careful about (and maybe independently verify) the pressure that accompanies various trial types. One might, for example, expect that decider trials early in a match may not contain as much felt pressure as decider trials that occur late in

Table 3: Estimation results for the fixed effects of the model fitted to data of single throws.

	Response variable:		
	checkout_new		
	(1)	(2)	(3)
CheckoutProportion	4.534***	4.562***	2.749
	(0.308)***	(0.310)***	(2.586)
	p = 0.000	p = 0.000	p = 0.288
CheckoutProportionOpp	0.076	0.084	−0.327
	(0.066)	(0.067)	(0.508)
	p = 0.253	p = 0.208	p = 0.520
Exp	0.005**	0.006**	−0.001
	(0.003)**	(0.003)**	(0.021)
	p = 0.035	p = 0.032	p = 0.962
Cb	0.150*	0.148*	0.710
	(0.082)*	(0.082)*	(0.658)
	p = 0.068	p = 0.074	p = 0.281
Constant	−2.394***	−2.408***	−1.570
	(0.129)***	(0.130)***	(1.041)
	p = 0.000	p = 0.000	p = 0.132
Observations	14,849	14,590	259
Note:			*p<0.1; **p<0.05; ***p<0.01

Table 4: Estimation results for the model including a *decider* covariate and a corresponding interaction term.

	Response variable:
	Checkout
CheckoutProportion	5.116***
	(0.057)
CheckoutProportionOpp	0.018
	(0.054)
Decider	0.039
	(0.224)
Exp	0.005
	(0.003)
Cb	−0.020
	(0.068)
CheckoutProportionOpp · Decider	−0.127
	(0.396)
Constant	−2.790***
	(0.062)
Observations	32,274
Note:	*p<0.1; **p<0.05; ***p<0.01

a match. This idea bears some relation to the Wells. et al. golf idea that pressure is maximized in golf when a player is close to the lead in the final round of a tournament (as opposed to earlier rounds). This suggests that the authors might want to look at the performance during various decider trials in a match – early, middle, late, and final (where, conceivably, one throw can win a match).

We would like to clarify our definition of decider trails. These deciders are always the last leg being played in the match. Here, the previous number of leg won by both contestants is equal and the winner of the last legs automatically wins the game. This is a win or go home leg. For a best-of-19 leg match, the decider leg is always the leg played when both players enter the leg with 9 legs won apiece. The winner of the decider leg wins the match 10-9. Hence, decider trails are always at the end of the match when both players can win the leg to win the match. As this was perhaps not sufficiently clear in the previous version of the paper, we added corresponding clarification in the revised paper.

8. The authors presented results across players. I have two thoughts about doing so. The first point concerns the magnitude of the coefficients for the individual players. Are any of them different enough from 0 that they fall out of the range expected from a random distribution? The point is that I wonder if it is necessary to name the players instead of labeling them with a meaningless descriptor. I know that other researchers in the area have explicitly avoided using real names to avoid the use of their results for the purpose of calling some players "chokers."

The random slopes for the performance in pressure situations do not improve the model fit, i.e. the coefficients are not "significant". In the revised version, we make this more clear by stating that the model without random slopes is preferred by the AIC. We still believe that it is worthwhile to present this additional analysis in the Appendix, as otherwise a reader may wonder as to whether there is any meaningful heterogeneity in the effect of pressure on performance. In addition, while we had thought that providing player names might be illustrative for readers familiar with the names, we understand the concern expressed here and hence have followed the suggestion to remove them from the paper.

9. One final point linking back to the social facilitation/social impairment issue. The application of that literature to the present task assumes that playing in front of an audience or with/against others applies "pressure". (The darts task may be seen as an example of the kinds of competitive/social tasks that are featured in that literature). Is that assumption plausible? That is, is that social pressure different from the kind of pressure that can come from non-social sources (e.g., playing for large monetary rewards)? The mechanisms posited for the social facilitation literature would probably suggest that the answer to this is "no", especially if one considers the mental interference produced by evaluation apprehension to be a special case of broad evaluative concerns. However, I know of colleagues who might try to make a case that there is a different kind of pressure involved if one is playing for a million dollars versus if one is playing to be acknowledged as the champion of one's city. This latter point leads to the possibility that clutch/choking may also be related to the nature or kind of pressure that is experienced during task performance. The authors, at their discretion, may wish to raise this point in their discussion.

Yes, pressure resulting from pure presence of others may differ from pressure due to playing for large monetary rewards. We mention this aspect in the discussion section of the revised paper.
