# Supplementary material for: Performance under pressure in skill tasks: An analysis of professional darts
Source: PLoS One. 2020 Feb 21;15(2):e0228870. doi: 10.1371/journal.pone.0228870 (PMC7034807; doi:10.1371/journal.pone.0228870)
Supplement: Attachment — Submitted filename: Plos One Darts Report.docx [file pone.0228870.s001.docx]

**Summary:** The authors employ throw-level data from professional darts tournaments over the course of one year to examine whether choking under pressure occurs in darts. Higher-pressure situations in this context are defined as those where a player’s opponent is likely to finish on their next turn, conditional on the player himself being within three darts of checking out, compared to situations where a player’s opponent is unlikely to finish. Choking, meanwhile, is defined as a decrease in the likelihood of checking out. By these definitions, the authors find that darts players actually perform better under pressure, i.e. that they are not susceptible to choking under pressure.

**Comments:**

I had two primary concerns with this paper, both of which are related to how choking under pressure in conceptualized in this context:

1. As noted in my summary above, the authors defined a high-pressure situation as one in which the win probability was lower (because the opponent was doing better), conditional on having a shot to win the leg, or checkout, that turn. However, I am concerned about the aptness of this definition, on which the paper’s conclusion of course relies. While it is difficult to provide an objective definition for a “high-pressure” situation bar, e.g., capturing biometric measures of stress, I would not think that pressure would be increasing in the opponent player’s chance of winning, conditional on having the opportunity to check out yourself. Consider, for instance, a case where a player has a 50% chance of winning compared to a case where a player has a 1% chance of winning; I would say that the first case is higher-pressure because “there’s more on the line to lose” (this could be framed in the context of expectations-based reference dependence, where the loss in utility following a lost opportunity to checkout is not as substantial when a player has a 1% chance of winning because their reference point was that they would fail to do so). In fact, defining a high-pressure situation as one in which a game is “close” is an established approach in the literature (Cao et al, 2011; Toma, 2017). Yet this is different from how the authors define choking in this paper, where the lower the chance a player has of winning, conditional on having a chance that turn, the higher the pressure, even when this means that the game is actually less “close” as in the example above. Since the evidence of choking under pressure presented here is of course contingent on the definition of a high-pressure situation, this naturally substantially affects my perspective on how the results ought to be interpreted.
2. A crucial assumption the authors must make in order to identify choking under pressure in this context is that all else is held constant when comparing play across situations in which the opponent is one dart, two darts, three darts, or more away from checking out. However, my understanding is that players may adjust their strategy according to their opponent’s position. For instance, if their opponent has no chance of checking out during their next turn, a player, especially one who needs either two or three throws to finish, might play more conservatively, throwing in a way that somewhat decreases their chance of checking out this round but positions them better for the following turn. This interpretation is exactly consistent with the results: When a player needs either two or three throws and their opponent cannot checkout their next turn, they are significantly less likely to checkout on the turn in question compared to a case where it is possible for their opponent to checkout; this difference is no longer significant when the player needs just one dart to checkout and thus conceivably has a more narrow strategy set. Thus, the higher probability of checking out on a given turn when the opponent has the chance to check out on their next turn seems quite conceivably due to the differences in optimal strategy across these cases rather than improved performance under pressure.

The ideal analysis would control for these differential strategies across cases (and this is why free throw or soccer penalty kicks are an appealing context in which to study choking – there is just one optimal strategy regardless of the position in the game: try to make the shot/goal). One possible approach would be to compare performance under these different scenarios (the darts required for self vs opponent) when the overall match is close vs not. If we observed that players were more likely to checkout when the overall match was close, conditional on the position they were in in a given lag, this would seem to provide more convincing evidence that darts players indeed excel under pressure. Footnote 7 suggests that no significant results were found using this analysis (which I would think should be highlighted!), though it was unclear whether this specific test was employed.

In addition to these two main concerns, noted below are strengths of the paper as well as some additional concerns and suggestions:

1. I appreciate that the authors took seriously the psychology literature that differentiates, for instance, the theoretical predictions of the impact of choking in effort- vs skill-driven tasks – this is an important literature to understand when exploring performance under pressure and the authors did this justice in the literature review. One suggestion, however, would be to avoid generalizing the findings in this paper to behavior in the labor market in the abstract and conclusion, as this is exactly an instance of crossing from a skill-driven to a (typically in the labor market) effort-driven task, and, as noted, the theory of choking under pressure would predict very different types of behavior across these domains.
2. The paper includes a section on why other studies may not have correctly identified choking under pressure. I indeed believe that this is an important section to include in order to credibly assert that the novel findings in this paper may be true across the board, but I had concerns about a number of the claims made:
   a. The authors argued that it’s difficult to disentangle pressure from “nuisance situations” such as weather conditions or the quality of the course. However, I see no reason to believe such nuisance conditions would be correlated with high-pressure situations, and so this should not pose a challenge to the identification of choking under pressure. Similarly, they argue that the small number of e.g. penalty kicks in soccer render the data sufficiently noisy as to make choking difficult to tease apart – I would see this as a specific concern if player fixed effects cannot be netted out (as in Dohmen, 2008) such that you may just have stronger players kicking in high-pressure situations, but otherwise a small number of observations alone should not invalidate results that have been found to be significant.
   b. The authors note that fatigue may enter in as high-pressure situations are those in which players are more likely to be fatigued because they have been playing longer or in a more intense game. Toma (2017), however, addresses this using free-throw data by showing (1) that choking only occurs in the final 30 seconds of a close basketball game and not before, when players are presumably similarly fatigued, and (2) that the number of seconds a player has played in a game does not predict choking.
   c. The authors argue that coaches may tend to put players best able to handle pressure in high-pressure situations, but this would seemingly work against the finding that players choke under pressure.
   d. The remaining points are specific only to certain types of analyses (e.g. soccer or experimental data) and thus cannot explain why findings across the board may not be valid.
3. These data seem like an interesting opportunity to look at heterogeneities in choking across players (once the right definition of choking is nailed down!), and while I appreciate that the authors took a step in this direction I think there’s more that could be done with the analysis:
   a. First, just a somewhat picky note that the authors’ claim is not seemingly supported by their evidence given that they do not have any data suggesting others may choke under pressure: “We argue that highly skilled individuals are able to digest pressure situations towards a positive outcome, while those who choke under pressure decide towards other professions.”
   b. I like the idea behind Figure 2, but I think I would have found more informative a graph with actual rather than predicted checkout probabilities across opponent scores for players with sufficient data; that way, not only the intercept would change, but we would actually see differences in choking susceptibility (this is seemingly a motive underlying *Model 2*, which I appreciate!). Perhaps in a new version of Figure 2 bins of different player “types,” by choking propensity, could be created and graphed.
4. The authors work with a great, fine-grained dataset, which is exactly what would be required for this type of analysis!

Some minor additional comments:

1. It took some time for the difference between Table 1 and Table 4 to sink in for me - My interpretation of the difference is that Table 1 looks at the probability of winning the leg overall while Table 4 looks at the probability of checking out *that turn*. This would be helpful to clarify, especially as the logic behind why the two are differentially relevant for the analysis was not made clear (this gets back to my second point – presumably the probability of winning a leg can’t be considered as a main outcome because this is dependent on opponent play, but even the strategy for the current turn is influenced by the opponent’s standing, so this still seems like a concern when considering checking out for a given turn).
2. For more visually-oriented folks, it may be helpful to plot the degree to which the changes in probabilities of checkouts correspond with the defined pressure in the situation; in other words, create a figure combining the information in Table 1 and Table 2 such that pressure is on the x axis and the probability of checking out is on the y axis.
3. I think there is no question that darts is a skill- rather than effort-driven task, so I did not find the discussion in Section 5 to be necessary. That said, some of the variables included in this analysis, e.g. the prize amount, would seemingly be strong proxies for the pressure in a situation (the higher the prize amount, the higher the pressure) and so could be incorporated in this way. The null effects of the controls alone suggest to me that, consistent with the null effects in the choking literature often found for e.g. more important tournament games, players may internalize more the pressure of making vs breaking a play at a given moment rather than any larger stakes of an overall game that wouldn’t be localized to a particular moment.
4. The statistical analysis tests for significant differences in checking out across opponent position only compared to the reference of the opponent not having the opportunity to check out the next turn; it would be useful to test for significant differences across the different opponent positions as well.
